# Screening and Quantification of the Synthetic Cannabinoid Receptor Agonist 5F‐MDMB‐PINACA From Seized Prison Paper Using Ultraperformance Liquid Chromatography–Mass Spectrometry Approaches

**DOI:** 10.1002/dta.3864

**Published:** 2025-02-07

**Authors:** Giorgia Vaccaro, Jacqueline L. Stair, Stewart B. Kirton, Daniel Baker, Amira Guirguis

**Affiliations:** ^1^ Department of Clinical, Pharmaceutical and Biological Science, School of Life and Medical Sciences University of Hertfordshire Hatfield UK; ^2^ Pharmacy Swansea University Medical School Swansea Wales UK; ^3^ School of Applied and Health Science London South Bank University London UK

**Keywords:** 5F‐MDMB‐PINACA, HPLC‐MS, new psychoactive substances, prison, synthetic cannabinoids

## Abstract

Infusing synthetic cannabinoid receptor agonists on paper quickly became a main route for illicit entry into prisons (i.e., mail correspondence) in the last decade. So far, there are limited data on validated detection methods and typical concentration profiles of these substances on paper to inform interventions. An approach to quantify and map the synthetic cannabinoid receptor agonist (SCRA) 5F‐MDMB‐PINACA on a seized paper sample from a UK Prison was determined using ultraperformance liquid chromatography–mass spectrometry. The seized paper sample was initially screened using ultraperformance liquid chromatography–quadrupole time‐of‐flight–mass spectrometry that confirmed the presence of 5F‐MDMB‐PINACA. A quantification method was then optimised and validated for the SCRA using a simple liquid chromatography–quadrupole Dalton–mass spectrometry system. Percentage recovery studies were carried out on paper spiked with 1, 5 and 20 mg/cm^2^ of 5F‐MDMB‐PINACA with five consecutive MeOH extractions. Results showed that one extraction recovered 83%–86%, while three extractions resulted in 98%–99% 5F‐MDMB‐PINACA recovery. Finally, the method was used to determine the concentration profile across the seized paper sample; 5F‐MDMB‐PINACA was in detectable amounts on all the paper subunits (*n* = 39) ranging between 0.26 and 55.13 μg/cm^2^, displaying a wide distribution of concentrations. Concentration profiles of SCRAs on seized paper samples are informative for interventions for those who are abusing or handling these substances in custodial settings but also provide key concentration ranges for those developing advanced methods of analysis in this area.

## Graphical abstract

Synthetic cannabinoid receptor agonists are being infused on paper and illicitly introduced into prisons through mail correspondence. An approach to quantify and map the synthetic cannabinoid 5F‐ADB on a seized paper sample from a UK prison was determined using ultraperformance liquid chromatography–quadrupole Dalton–mass spectrometry.

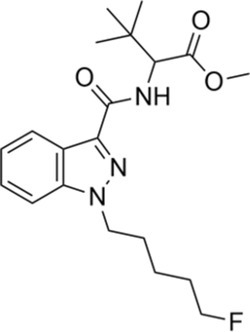

## 1 Introduction

A recent literature review suggests that the abuse of synthetic cannabinoid receptor agonists (SCRAs) in custodial settings has increased over the last 10 years [1]. SCRAs are one of the most widely abused new psychoactive substances (NPS) that mimic the effect of phytocannabinoids and target the CB_1_ and CB_2_ receptors in the brain and other organs. The classification of SCRAs varies significantly based on their chemical structure, with an estimated 361 distinct SCRAs reported worldwide [2]. While in 2023, 254 SCRAs were being monitored in the EU alone [3]. Given the wide range of SCRAs available (e.g., classical cannabinoids, iHybrid and aminoalkylindoles), research into their pharmacology and toxicology remains limited [4]. Several studies have reported that SCRAs are the most widely used NPS in custodial settings [1, 5]. A cross‐sectional study in a UK male prison population reported that 46.7% of participants had used a SCRAs mainly in paper‐based preparations and via e‐cigarette cartridges [6]; their misuse in this scenario was linked to greater psychological distress. Duke et al. linked 48% of drug‐related deaths from 2015 to 2020 in English and Welsh prisons to SCRA abuse [7]. A systematic review by Chiappini et al. reported on the use and psychiatric implications of NPS in custodial settings and found SCRAs to be the most reported NPS using both observational studies and case series [5]. They highlighted several SCRAs that dominated the market before 2018 (i.e., 5F‐MDMB‐PINACA aka 5F‐ADB, AB‐FUBINACA, ADB‐FUBINACA and AMB‐FUBINACA) and those more recently (i.e., 5F‐MDMB‐PICA, 4F‐MDMB‐BINACA and MDMB‐4en‐PINACA). Certain SCRAs, such as 5F‐MDMB‐PINACA, appeared to have longevity across the different time periods. Vaccaro et al. also reviewed the use of NPS in these settings with a focus on drug forms and detection methods [1]. Once again, SCRAs were the most reported NPS, including substances like 4F‐MDMB‐BINACA, MDMB‐4en‐PINACA and 5F‐MDMB‐PINACA, primarily found infused on paper matrices [1]. More recently, a study in English prisons analysed 1250 paper seizures and found 52% of the seizures contained at least one SCRA, with the most detected being 5F‐MDMB‐PINACA [8]. Additionally, a study carried out in Brazilian prisons reported seven SCRAs detected on infused paper where 5F‐MDMB‐PICA (36.4%) and MDMA‐4en‐PINACA (24.1%) were the most prevalent [9]. Although 5F‐MDMB‐PINACA was the predominant SCRA in the market until late 2018, it continued to be reported in the literature and in a recent study by Giorgetti et al., alongside the more recent SCRA MDMB‐4en‐PINACA [10]. It must be noted that other NPS, such as novel synthetic opioids (NSO) and novel benzodiazepines (NBZs), are also starting to circulate in European custodial settings either in the form of infused letters or in the new form of waxy‐ or putty‐like materials highlighting a possible change in trends [10, 11]. To overcome the smuggling of infused letters some UK prisons started photocopying prisoners' correspondence, this approach proved time consuming and, at times, complex. Confidential correspondence can only be opened and inspected under specific conditions, as outlined by ‘Rule 39’ in English and Welsh prisons and ‘Legal Mail’ regulations in Scotland, complicating routine checks and photocopying efforts [1]. Similar approaches have been taken by the United States, yet full international compliance will take time [12]. Thus, the use of paper matrices to smuggle NPS into custodial settings continues to be a source of entry, and developing detection methodologies specifically targeting NPS on paper media is still relevant.

Preliminary tests for detecting SCRAs are quick and simple methods, lacking accuracy, used to check if such substances might be present before performing confirmatory analysis. Common tests include colourimetric tests, thin layer chromatography and immunoassays. A review of the confirmatory methods used for SCRA‐infused paper analysis in prisons showed that gas chromatography–mass spectrometry (GC–MS) and high‐performance liquid chromatography/ultraperformance liquid chromatography–mass spectrometry (HPLC/UPLC‐MS), for example, time‐of‐flight (ToF) or orbitrap, were the main techniques used. However, there were a range of solvents (i.e., methanol [MeOH], methanol:water [MeOH:H_2_O] [50:50] and dichloromethane:methanol [DCM:MeOH] [75:25]), extraction approaches (i.e., ultrasonication and vortex mixing) and time parameters (i.e., 5–30 min) reported across studies [1, 8]. The majority of these studies were qualitative, to determine the presence or absence of SCRAs, which is often what is required for forensic analyses. However, quantitative studies are needed to determine typical concentration ranges, which are essential for validating extraction methods, guiding the development of novel detection methodologies, for example, infield point‐of‐care testing, and informing dose and exposure parameters for harm reduction and intervention strategies.

Currently, there are limited studies regarding the typical concentration ranges and/or the concentration profiles across SCRA‐infused paper seized from prisons. Norman et al. were the first to report on the quantitative results using GC–MS of SCRA‐infused papers, seized in prison, reporting a range of < 0.05–1.17 mg/cm^2^ and mapping profile ranges for AMB‐CHMICA (0.47–2.38 mg/cm^2^) and 5F‐MDMB‐PICA (0.48–1.34 mg/cm^2^) [13]. Giorgetti et al. quantified a single letter impregnated with MDMB‐4en‐PINACA (77 μg/cm^2^) and 5F‐MDMB‐PINACA (6.5 μg/cm^2^) additionally detecting AP‐237 (1.2 μg/cm^2^) highlighting the potential spread of NSO‐infused letters in prisons [10]. In an effort to understand the concentration profiles across SCRA‐infused paper, Angerer et al. examined different infusing papers and drying techniques by preparation of simulated paper samples [14]. These data are key for the development and application of qualitative presumptive tests. For example, ion mobility spectrometry (IMS) for SCRAs on paper has been shown to achieve detection limits of 0.5–1000 and 0.7–3.6 ng for a range of 25 SCRAs [15]. More recently, Cozier et al. developed an ultraportable device to detect SCRAs on physical matrices (i.e., paper, fabric and herbal material) based on emission spectra in which a SCRA's limit of detection (LOD) was estimated at 10 mg/cm^2^ [16]. Thus, both IMS and emission spectroscopy techniques demonstrate good sensitivity for detecting SCRAs on paper, but further investigations are necessary due to the limited existing data.

This short paper aims to advance the understanding of quantitative data regarding SCRAs on paper matrices, focusing on 5F‐MDMB‐PINACA as the representative SCRA, which has been frequently reported in custodial settings and detected on a seized paper sample. We explored a quantitative method using ultraperformance liquid chromatography–quadrupole Dalton–mass spectrometer (UPLC‐QDa‐MS) to quantify the amount of 5F‐MDMB‐PINACA on paper, after identification performed using ultraperformance liquid chromatography–quadrupole ToF–mass spectrometer (UPLC‐QToF‐MS) (Table S1 and Figure S1). Furthermore, we conducted recovery studies across a range of concentrations, as well as a concentration profile analysis in a prison seized paper sample.

## 2 Material and Methods

### 2.1 Chemicals and Reagents

5F‐MDMB‐PINACA reference standard (RS) (99% purity) was obtained from Chiron AS (Trondheim, Norway). HPLC grade MeOH and common grade acetone (99% purity) were obtained from Fisher Scientific (Loughborough, UK). Ultrahigh purity Millipore Water (MW) (18 MΩ/cm) was obtained from Milli‐Q water purification system (Merck, UK). Common A4 printing 80‐g/m^2^ density paper sheet (Type 1: Envirocopy A4 500 sheet; ECF) was employed for the preparation of simulated paper samples.

### 2.2 Seized Paper Sample

A nonjudicial sample, A5 paper sheet (148 × 210 mm), was seized by Her Majesty Prisons Service (HMPS) supplied in tamperproof polythene evidence bag in October 2018 and analysed in April 2019. A presumptive IMS analysis performed by the prison officers indicated the presence of SCRAs as ‘Spice’ which did not specify the exact substance. Evidence bags were coded and recorded as per standard operating procedures and UK legislation. The seized paper sample was divided into roughly 316 units (*N*) of 1 cm^2^ using a sampling grid and scalpel.

### 2.3 5F‐MDMB‐PINACA Quantification

A Waters Acquity UPLC coupled to a QDa‐MS (Milford, MA, USA), running under MassLynx V.4.2, was employed to quantify 5F‐MDMB‐PINACA present in the seized paper sample. Mobile phases used were (A) MW with 0.1% v/v formic acid and (B) MeOH with 0.1% v/v formic acid. The gradient used was 0.0–0.5 min: 25% (B) hold, 0.5–3.5 min 25%–90% (B), 3.5–6.0 min 90%–95% (B), 6.0–6.5 min 95%–50% (B), 6.5–7.0 min 50%–75% (B) and then hold for 2 min. A flow rate of 0.6 mL/min and a column temperature of 30°C were employed with a Phenomenex Kinetex C_18_ column (100 Å 100 × 2.1 mm × 2.6 μm particle size) and SecurityGuard ULTRA UPLC holder and cartridge C_18_ 2.1 mm, sourced from Phenomenex (Macclesfield, UK). 5F‐MDMB‐PINACA was monitored in positive mode [M + H] (378.5 *m/z*) in a selected ion recording (SIR) mode and quantified using a standard curve.

#### 2.3.1 Method Validation

The UPLC method was validated for 5F‐MDMB‐PINACA using the UNODC guidance [17] for the following parameters: system suitability, linearity and working range, LOD and limit of quantification (LOQ), precision under repeatability and reproducibility conditions, accuracy and recovery. Six calibration standards (1–50 μg/mL) were prepared for the quantification of 5F‐MDMB‐PINACA and to calculate the linearity as part of the method validation (Table S2). Six replicate injections per concentration were evaluated.

#### 2.3.2 Simulated Paper Analysis

Simulated paper samples were prepared to evaluate the percentage recovery of 5F‐MDMB‐PINACA. Five 1‐cm^2^ replicate samples were prepared each at three concentrations using common printing paper, and five consecutive extractions were performed on each sample. For the highest concentration, 20 μL of 1‐mg/mL solution was pipetted onto a 1‐cm^2^ piece of paper (~20 μg/cm^2^). While for the middle and lowest concentrations, 50 and 10 μL of a 0.1‐mg/mL solution were pipetted onto 1‐cm^2^ pieces of paper (~5 and 1 μg/cm^2^, respectively). The 5F‐MDMB‐PINACA solutions were pipetted onto paper while suspending them by a pair of tweezers between a clamp. The simulated paper samples were allowed to dry completely in the same position to avoid any loss. Each sample was placed in a separate reaction tube with 1 mL of MeOH, sonicated and centrifugated for 10 and 5 min, respectively. This process was repeated five times using fresh MeOH. Each extract was transferred into an HPLC vial and analysed separately using the UPLC‐QDa‐MS. The total % recovery of 5F‐MDMB‐PINACA was obtained by summing up individual % recoveries determined for each extract. A preliminary examination of the paper matrix effect on the quantification was also carried out (Figure S2 and Table S3).

#### 2.3.3 Seized Paper Analysis

**FIGURE 1:**
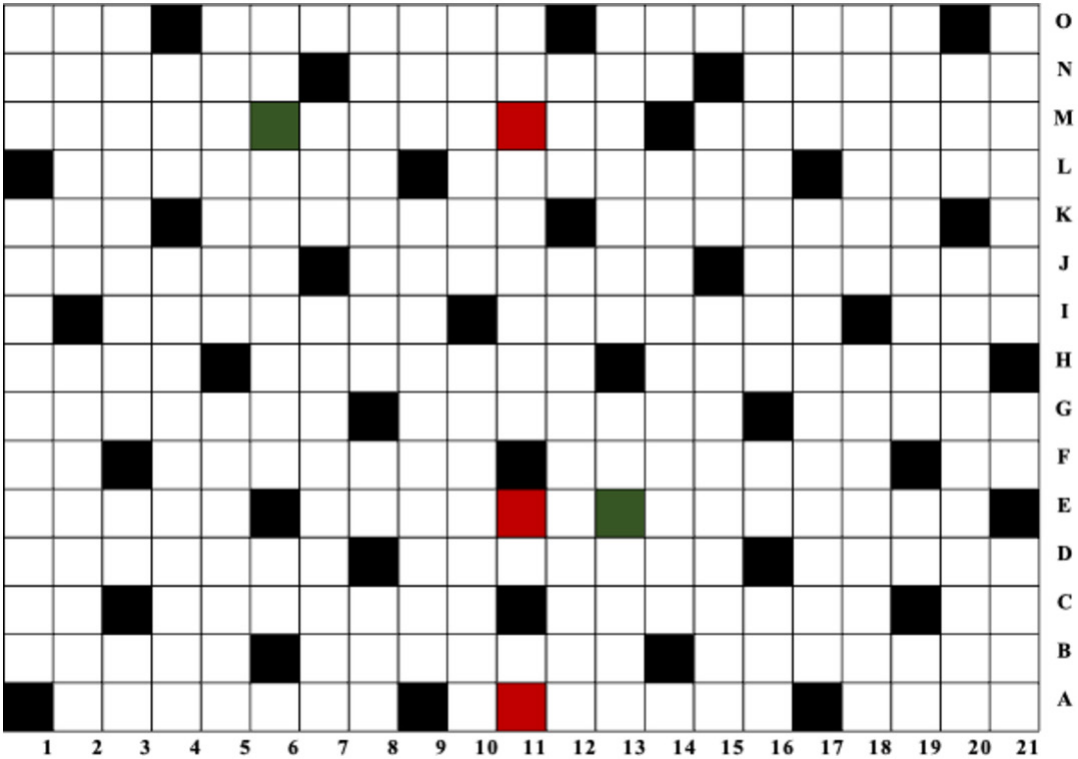
Sampling grid for the prison paper sample in which the red subunits were used for the screening analysis, the green subunits were the sampling starting points and the black subunits were used for the 5F‐MDMB‐PINACA quantification.

To establish the sample size on which to perform the quantification, a statistical frequentist approach using the hypergeometric distribution was employed to determine the number of subunits used to analyse and achieve representative sampling [17, 18]. The frequentist approach was chosen as it assumes that a fixed but unknown proportion of the seizure contains drugs. To guarantee with 99% confidence that at least 90% of the subunits contain illicit substances, 39 (*n*) subunits were sampled [19]. Each sample was taken at an interval of eight subunits with two random subunits, 13E and 6M, coloured in green (Figure 1), selected as starting points using the random function on Excel. For the extraction, the method described in Section 2.3.2 was employed; however, in this case, only three consecutive extracts were evaluated.

## 3 Results and Discussion

### 3.1 5F‐MDMB‐PINACA Quantification

#### 3.1.1 Method Validation

A UPLC‐QDa‐MS method was optimised and validated according to the UNODC [17]. The retention time of 5F‐MDMB‐PINACA was 3.99 ± 0.05 min. Linearity *r* ^2^, LOD and LOQ values calculated on six calibration curves were found to be 0.998 ± 0.001, 0.059 ± 0.027 ng/mL and 0.18 ± 0.08 ng/mL, respectively. All parameters evaluated for the UPLC‐QDa‐MS method validation of 5F‐MDMB‐PINACA were found to be within the acceptance criteria (Table S4). Thus, the method was used for the subsequent analysis of the paper samples.

#### 3.1.2 Simulated Paper Analysis

4F‐MDMB‐PINACA was chosen as the focus because it was the specific SCRA initially detected on the seized prison paper analysed and reported in recent studies [10, 20]. This compound was deemed suitable as a representative for the simulated paper analysis and the development of the quantification method due to its structural similarity to other SCRAs more recently found in custodial settings [20, 21], such as MDMB‐4en‐PINACA, 5F‐MDMB‐PICA and 4F‐MDMB‐BINACA. Table 1 highlights the structural similarities between these SCRAs. By using 5F‐MDMB‐PINACA as a representative compound, the methodology can be adapted for application to additional real‐world samples containing related SCRAs. Recovery of 5F‐MDMB‐PINACA was calculated for three different deposited amounts (i.e., 1, 5 and 20 mg/cm^2^) using five replicates for each concentration. After three consecutive MeOH extractions, the quantity of 5F‐MDMB‐PINACA extracted was 98.4% ± 0.6%, 98.9% ± 0.6% and 98% ± 1% for 1, 5 and 20 mg/cm^2^, respectively (Table 2). The concentration of the 5F‐MDMB‐PINACA extracted after the fourth and fifth extraction was below the LOQ. The finding is in general agreement with Norman et al., who verified that three consecutive extractions were sufficient to quantitatively extract (i.e., 100% recovery) SCRAs at the higher concentration of 75 mg/cm^2^ from paper [13]. The discrepancies between the % recovery could be due to the different solvent employed by Norman et al., 75:25 DCM:MeOH which might have played a role in enhancing the extraction of the SCRAs because their solubility is higher in solvents with low polarity [13]. In contrast to this speculation, several studies have employed MeOH as the extraction solvent for SCRA‐infused papers that were seized. Angerer et al. extracted each 1‐cm^2^ subunit with MeOH (2 mL) undergoing 15 and 20 min of ultrasonication and centrifugation, respectively, for each of the three consecutive extractions performed. The extracts were then combined and analysed using HPLC‐DAD instrument [14]. Furthermore, Giorgetti et al. extracted a total paper surface of 975.7 cm^2^ cut in 5‐cm^2^ pieces by adding 9 mL of MeOH under gentle mixing for 10 min, to semiquantify SCRAs on a letter seized in a German prison [10]. Specifically, Norman et al. calculated the % recovery of the analyte on simulated samples [13] giving information on the efficiency of the solvent employed. Nevertheless, for rapid qualitative assessment, the use of MeOH for a single extraction seems sufficient for similar indazole‐like substances; Avci Akca et al. implemented a 50:50 MeOH:H_2_O extraction solvent with 30‐min vortex mixing for a qualitative assessment of 1250 seized paper samples using LC‐QToF‐MS [8].

**TABLE 1:** Structural differences between 5F‐MDMB‐PINACA, MDMB‐4en‐PINACA, 5F‐MDMB‐PICA and 4F‐MDMB‐BINACA. [Correction added on 10 June 2025, after first online publication: 5F‐MDMB‐BINACA, Indazole, Valine‐derived (MDMB), 4‐En‐butyl and 5‐Fluoropentyl were updated accordingly.]

Groups	5F‐MDMB‐PINACA	MDMB‐4en‐PINACA	5F‐MDMB‐PICA	4F‐MDMB‐BINACA
Core	Indazole	Indazole	Indole	Indazole
Linker	Carboxamide	Carboxamide	Carboxamide	Carboxamide
Head	Isoleucine‐derived (MDMB)	Isoleucine‐derived (MDMB)	Isoleucine‐derived (MDMB)	Isoleucine‐derived (MDMB)
Tail	5‐Fluoropentyl	Pent‐4‐enyl	5‐Fluoropentyl	4‐Fluorobutyl

**TABLE 2:** Percentage recoveries for 5F‐MDMB‐PINACA on simulated paper samples (1 cm^2^).

5F‐MDMB‐PINACA deposited (mg/cm^2^)	1st extraction %	2nd extraction %	3rd extraction %	4th extraction %	5th extraction %	Total % recovery
1	84 ± 2	14 ± 2	0.6 ± 0.3	< LOQ	< LOQ	98.4 ± 0.6
5	83 ± 4	15 ± 3	1.6 ± 0.3	< LOQ	< LOQ	98.9 ± 0.6
20	86 ± 3	11 ± 2	1.0 ± 0.5	< LOQ	< LOQ	98 ± 1

#### 3.1.3 Seized Paper Analysis

**FIGURE 2:**
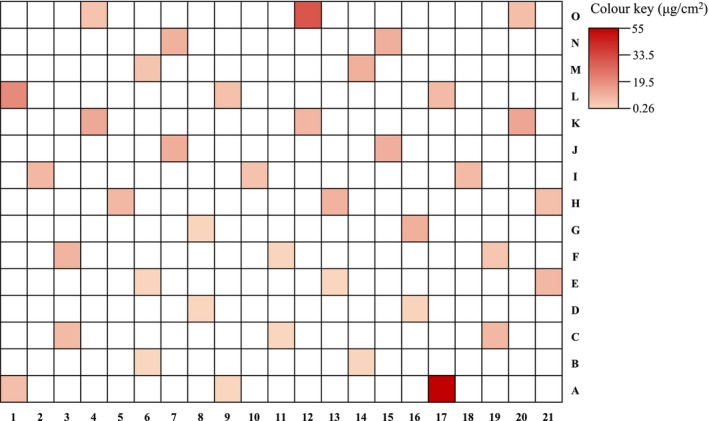
5F‐MDMB‐PINACA gradient across the sampling subunits of the seized paper sample ranging from 0.26 μg/cm^2^ for subunit 11C to 55 μg/cm^2^ for subunit 17A. [Correction added on 10 June 2025, after first online publication: Figure 2 has been replaced with the revised version to include the colour key.]

The quantification of 5F‐MDMB‐PINACA on the 39 prison paper subunits, after three consecutive extractions, was carried out (Table 3). This yielded a mean concentration of 8.27 ± 9.83 μg/cm^2^ (RSD 119%) and a median of 7.28 μg/cm^2^ with a range of 0.26–55.13 μg/cm^2^, showing a significant degree of variability across the seized paper. The amount of 5F‐MDMB‐PINACA quantified on the seized paper in this study was two to three orders of magnitude lower than previously reported in the literature < 0.05–1.17 mg/cm^2^ for one study [13] and in the same range of 6.5 μg/cm^2^ for a more recent study [10], thus indicating that analytical methods need to have a large dynamic range. It is noteworthy that Angerer et al. reported a relatively low inhomogeneity of distribution (22% RSD) of synthetic cannabinoids across the paper, which contrasts with our findings of a much higher variability (119% RSD). This discrepancy highlights the need for careful consideration of sampling and extraction methods when analysing seized materials. Combining the extracts resulting from consecutive extractions offers the advantage to accurately measure the total recovery of the analyte, as opposed as analysing them separately where some of the subsequent extracts may contain the analyte in a concentration below the instrument LOQ. All subunit extract concentrations were above the LOD, indicating the suitability of using a UPLC‐QDa‐MS for SCRA quantification on paper once identity is confirmed. Selective mapping of the 5F‐MDMB‐PINACA content across the seized paper sample was performed. The sampling strategy employed a statistical approach to gain knowledge on the concentration of the SCRAs while considering budget and time constraints. The 5F‐MDMB‐PINACA concentrations determined in the 39 selected sampling subunits are shown as a gradient in Figure 2. In general, the 5F‐MDMB‐PINACA content is greater at the paper edges and in particular along the top subunits from J to O as well as the right‐hand side subunits from 17–21. The selective mapping provided a good representation of the SCRA estimated content across the paper and may be a strategy to use for future studies to reduce analysis time when quantitatively assessing drugs on paper.

**TABLE 3:** 5F‐MDMB‐PINACA concentration on 39 subunits of the prison paper sample.

Subunits	5F‐MDMB‐PINACA on subunits, AVE ± STD μg/cm^2^ (%RSD)
1A	6.19 ± 0.09 (1.4%)
1L	19.69 ± 0.07 (0.35%)
2I	8.10 ± 0.03 (0.36%)
3C	7.28 ± 0.08 (1.1%)
3F	9.19 ± 0.06 (0.68%)
4K	11.97 ± 0.09 (0.72%)
4O	5.14 ± 0.09 (1.8%)
5H	8.05 ± 0.07 (0.93%)
6B	0.41 ± 0.01 (1.4%)
6E	0.909 ± 0.002 (0.17%)
6M	5.00 ± 0.09 (1.8%)
7J	10.8 ± 0.2 (1.6%)
7N	9.93 ± 0.03 (0.32%)
8D	0.372 ± 0.003 (0.89%)
8G	0.61 ± 0.01 (0.90%)
9A	0.349 ± 0.001 (0.31%)
9L	5.8 ± 0.1 (1.7%)
10I	5.30 ± 0.06 (1.1%)
11C	0.2591 ± 0.0005 (0.21%)
11F	0.68 ± 0.01 (1.1%)
12K	8.48 ± 0.07 (0.88%)
12O	33.25 ± 0.02 (0.55%)
13E	0.378 ± 0.005 (1.3%)
13H	9.5 ± 0.1 (1.1%)
14B	0.73 ± 0.01 (0.93%)
14M	10.23 ± 0.04 (0.41%)
15J	10.3 ± 0.1 (1.1%)
15N	8.78 ± 0.06 (0.66%)
16D	1.20 ± 0.01 (1.1%)
16G	10.09 ± 0.03 (0.30%)
17A	55.1 ± 0.1 (0.22%)
17L	7.4 ± 0.1 (1.7%)
18I	5.08 ± 0.02 (0.42%)
19C	7.98 ± 0.04 (0.56%)
19F	4.45 ± 0.03 (0.60%)
20K	12.8 ± 0.3 (1.3%)
20O	6.52 ± 0.09 (1.4%)
21E	8.15 ± 0.05 (0.65%)
21H	5.9 ± 0.1 (1.9%)

Based on the results of the paper analysis, inferences on the paper drying method, presumptive testing and dosing were made. The content distribution of the SCRAs on the paper sometimes could indicate the way in which the paper was dried. Angerer et al. found that using a flat and smooth surface to dry the SCRA‐infused paper resulted in a relatively low inhomogeneity of distribution across the paper [14]. However, it was not possible to speculate as to whether the seized paper examined in this study was dried by hanging up the paper or laying it flat on a surface, as only the results of the quantification of the SCRA using one type of drying technique were included [14]. However, Norman et al. presented a more comprehensive controlled SCRA paper dosing experiment for both drying techniques in triplicate, for 5F‐MDMB‐PINACA [13]. Thus, it could be speculated that the seized paper in this study was dried flat, where higher concentration areas were localised peripherally and not exclusively at the bottom [13]; however, the 119% RSD found among our sample's subunits is in contrast with this theory. The high variability of the SCRA quantity across the seized paper demonstrates that the process employed to prepare such samples is neither controlled nor consistent, hence does not ensure uniform distribution of the drug on paper. In terms of presumptive testing, IMS would be suitable for the seized paper sample when sampling the paper as a whole based on reported SCRA LODs [15, 16], as the maximum 5F‐MDMB‐PINACA concentration detected on the sized paper was 55 μg/mL. In contrast, this concentration falls below the estimated LODs reported by Cozier et al. for the ultraportable emission spectra device. Concerning the use of SCRA‐infused papers in prisons, before the smoking ban in United Kingdom, inmates reportedly smoked SCRA‐infused paper by adding small pieces to cigarettes. Since the ban, they have adapted by placing the SCRA‐infused paper between the heating element and e‐liquid cartridge of vaping devices [8, 13]. Additionally, anecdotal reports indicate other routes of administration, such as licking, chewing, swallowing or placing small pieces of SCRA‐infused letters in the eye [1, 8]. Angerer et al. reported that smoking a piece of paper with an area of 0.5–1 cm^2^ would lead to a dose comparable to doses proposed for MDMB‐CHMICA in internet fora (0.05–0.3 mg) and therefore would be sufficient to produce an effect [14]. Specific information on the proposed dose for our SCRA of interest were not found in the literature. However, given that 5F‐MDMB‐PINACA is more potent than MDMB‐CHMICA [20], even a lesser amount could be needed to achieve the desired psychoactive effects. In our study, 5F‐MDMB‐PINACA was quantified and found to be 55.13 μg/cm^2^ in the upper end corresponding to an amount which could have an effect. Consistently, Norman et al. found among their seized samples 1‐cm^2^ precut subunits or slightly smaller circular units, alone or in combination with e‐cigarettes, suggesting the size of a typical dose [13]. Due to uneven distribution on the paper and the different potency of the SCRAs employed, the psychoactive effects can vary greatly and be unpredictable.

## 4 Conclusions

The SCRA 5F‐MDMB‐PINACA was identified, and the quantity selectively mapped across the 39 subunits of a seized paper sample from HMPS. Due to the rapidly changing nature of SCRA use, 5F‐MDMB‐PINACA was deemed a suitable representative compound for other emerging structurally related SCRAs such as MDMB‐4en‐PINACA. A method using a UPLC‐QDa‐MS system was validated and appropriate for quantification of the SCRA. Extraction from paper using MeOH was sufficient for obtaining > 98% recovery with three consecutive extractions, while > 83% recovery was obtained with only one extraction. Thus, for qualitative assessment, one extraction is adequate but likely enhanced with different extraction solvents. This will also depend on the analysis method used, where a MeOH extraction solvent can be preferred for LC–MS systems. The extraction method showed consistent results for 5F‐MDMB‐PINACA infused at 1‐, 5‐ and 20‐mg/cm^2^ paper. The selective mapping approach saved time yet still demonstrated high variability of the SCRA concentration across the seized paper sample (RSD 119%). Thus, analysis methods of SCRAs on paper should use multiple sampling areas and ensure a large dynamic range for detection purposes. More recently, reductions in SCRA‐infused paper seized is being reported due to photocopying of prisoner's mail; however, ‘confidential correspondence’ and full international compliance still present challenges. Thus, future work should focus on evaluating a wider range of newly emerging SCRAs, for example, MDMB‐4en‐PINACA and MDMB‐INACA; other NPS classes for example, NSO and NBZs; and their mixtures starting to circulate in custodial settings [10, 11], as well as novel smuggling methods employed, such as the use of waxy‐ or putty‐like materials concealed in vape pods [11].

## Conflicts of Interest

The authors declare no conflicts of interest.

## Supporting information


**Table S1.** Entries reported on HighResNPS.com corresponding to 378.2187 *m/z*.
**Figure S1.** MS of subunits 11M (a) and 5F‐MDMB‐PINACA RS (b) and blank paper extract in MeOH (c).
**Table S2.** UPLC‐PdA‐QDa‐MS 5F‐MDMB‐PINACA calibration standards dilution scheme.
**Figure S2.** Different types of paper matrix evaluated for the study.
**Table S3.** Summary of results of the five paper types evaluated in the paper matrix evaluation study.
**Table S4.** Method validation summary results.

## Data Availability

The data that support the findings of this study are available from the corresponding author upon reasonable request.
