# Supplementary material for: Screening and Quantification of the Synthetic Cannabinoid Receptor Agonist 5F‐MDMB‐PINACA From Seized Prison Paper Using Ultraperformance Liquid Chromatography–Mass Spectrometry Approaches
Source: Drug Test Anal. 2025 Feb 7;17(9):1607–13. doi: 10.1002/dta.3864 (PMC12401647; doi:10.1002/dta.3864)
Supplement: Supplementary file 1 — Table S1. Entries reported on HighResNPS.com corresponding to 378.2187 m/z. Figure S1. MS of subunits 11M (a) and 5F‐MDMB‐PINACA RS (b) and blank paper extract in MeOH (c). Table S2. UPLC‐PdA‐QDa‐MS 5F‐MDMB‐PINACA calibration standards dilution scheme. Figure S2. Different types of paper matrix evaluated for the study. Table S3. Summary of results of the five paper types evaluated in the paper matrix evaluation study. Table S4. Method validation summary results. [file DTA-17-1607-s001.docx]

**Supporting Information**

Screening and Quantification of the Synthetic Cannabinoid Receptor Agonist 5F-MDMB-PINACA from Seized Prison Paper Using Ultra-Performance Liquid Chromatography-Mass Spectrometry Approaches

Giorgia Vaccaro^1^, Jacqueline L. Stair^1^, Stewart B. Kirton^1,3^, Daniel Baker^1^ and Amira Guirguis^1,2^ *

*Corresponding Author (e-mail address amira.guirguis@swansea.ac.uk)

^1^Department of Clinical, Pharmaceutical and Biological Science, School of Life and Medical Sciences, University of Hertfordshire, Hatfield, UK AL10 9AB

^2^ Pharmacy, Swansea University Medical School, The Grove, Singleton Campus, SA2 8PP, Swansea, Wales, UK

^3^ School of Health and Care Science, College of Health and Science, University of Lincoln, Brayford Pool, Lincoln LN6 7TS

**1. SCRAs identification using UPLC-QToF-MS**

**Methodology**

A Waters Acquity^®^ UPLC coupled to a Waters Xevo G2S QtoF-MS (Milford, MA, USA), running under MassLynx V.4.2 was employed to screen for SCRAs present in the seized paper sample. The method was adapted from Von Cüpper et al. (2021) using a Waters Acquity UPLC HSS T3 C18 column (150 x 2.1 mm x 1.8 µm particle size)^1^. The mobile phases used were (A) 5 mM of aqueous ammonium formate buffer, pH 3 (formic acid) and (B) acetonitrile with 0.1% v/v formic acid. The gradient was 0-0.5 min: 13% (B) hold, 0.5-10 min: 13 to 50% (B), 10-10.75 min: 50 to 95% (B), 10.75-12.25 min: 95% (B) hold, 12.25-15 min: 95 to 13% (B) and from 15-20 min: 13% (B) hold. A flow rate of 0.4 mL/min and a sample volume of 5 µL was employed with a desolvation temperature of 400 °C; source temperature of 150 °C, and a capillary voltage at 0.8 kV. The QtoF was operated in the Data Independent Acquisition (DIA) MS^E^ employing collision energies ranging from 0 to 40 eV in both resolution and sensitivity modes. Further fragmentation spectra were acquired in MS/MS Selected Reaction Monitoring (SRM) on the precursor ion of interest. A blank (pure MeOH) and

quality control samples (APINACA and 5F-MDMB-PINACA RS at 500 ng/mL) were run at the start and end to evaluate method performance.

**Sample preparation**

Three subunits (1 cm^2^) were cut from the bottom (11A), middle (11E), and top (11M) of the seized paper sample (Figure 1) to determine the presence of any SCRAs and for a concentration estimate. To extract the analytes from the paper, each subunit was placed in a reaction tube with 0.5 mL MeOH. The reaction tubes were sonicated and centrifugated for 10 and 5 min, respectively. This process was carried out three times using fresh MeOH, the extracts were combined, dried down, reconstituted in 0.5 mL of MeOH and analysed. The same procedure was applied to a blank paper (BP) sample. The online liquid chromatography-high resolution mass spectrometry (LC-HRMS) HighResNPS.com database was used to identify potential SCRAs with an m/z tolerance of 5 ppm. A direct comparison between the spectra of the identified substance (5F-MDMB-PINACA) and its certified RS was performed.

**Results**

The TIC, in both resolution and sensitivity mode of the top (11M), middle (11E), bottom (11A) subunits, the BP extract and the blank MeOH samples were compared. The TIC’s showed the presence of one peak at 11.86 min with a *m/z* value of 378.2206 in each seized paper sample analysed. A database search (HighResNPS) revealed nine positional isomers corresponding to this ion (Table SI1).

**Table SI1.** Entries reported on HighResNPS.com corresponding to 378.2187 m/z.

| **Compounds** | **Molecular formula** | **P mass** | **F1 mass** | **F2 mass** | **F3 mass** | **F4 mass** | **F5 mass** | **F6 mass** |
| --- | --- | --- | --- | --- | --- | --- | --- | --- |
| 2F-MDMB-PINACA | C_20_H_28_FN_3_O_3_ | 378.2187 | 233.1085 | 318.1976 | 145.0396 | - | - | - |
| 3F-MDMB-PINACA |  |  | 233.1085 | 318.1976 | 145.0396 | - | - | - |
| 4F-EDMB-BUTINACA |  |  | - | - | - | - | - | - |
| 4F-MDMB-PINACA |  |  | 233.1085 | 213.1022 | 145.0396 | 69.0699 | - | - |
| 5F-MDMB-PINACA |  |  | 233.1085 | 318.1976 | 213.1022 | 145.0396 | 251.119 | 69.0699 |
| 5F-AEB |  |  | 233.1085 | 213.1022 | 304.1819 | 251.119 | 145.0396 | 69.0699 |
| 5F-MDMB-PINACA 2'-indazole isomer |  |  | - | - | - | - | - | - |
| 5F-MDMB-P4AICA |  |  | 233.1085 | - | - | - | - | - |
| 5F-MDMB-P7AICA |  |  | 233.1085 | 318.1976 | 145.0396 | - | - | - |

Injection of 5F-MDMB-PINACA RS confirmed the identity of this peak by matched retention time (RT), parent ion *m/z,* and fragmentation pattern. The RT and the protonated parent ion of 5F-MDMB-PINACA RS resulting from the MS^E^ method were 11.86 min and 378.2191 *m/z*, respectively, in line with our findings (± 3.96 ppm). A comparison of the MS/MS SRM data of the three seized paper subunits showed the following common fragment ions 233.1086, 318.1980, 213.1018, 145.0394, 177.0454 *m/z, only the* MS of the bottom subunit (11M) has been presented in Figure S1a. While the following fragment 233.1088, 318.1985, 213.1017, 145.0393, 177.0453 *m/z* resulted from the 5F-MDMB-PINACA RS (Figure S1b) analysis. Additionally, the MS of a blank paper extract in MeOH subunits is presented in Figure S1c A discrepancy of < 5ppm was found for each fragment ion, which was the criterion used for identification with the relative abundance. Given that the identification of the MDMB-5F-PINACA was based on a mass spectral match and the purchase of the RS showing the same retention time, the analyte detected could also be an isomer of MDMB-5F-PINACA.


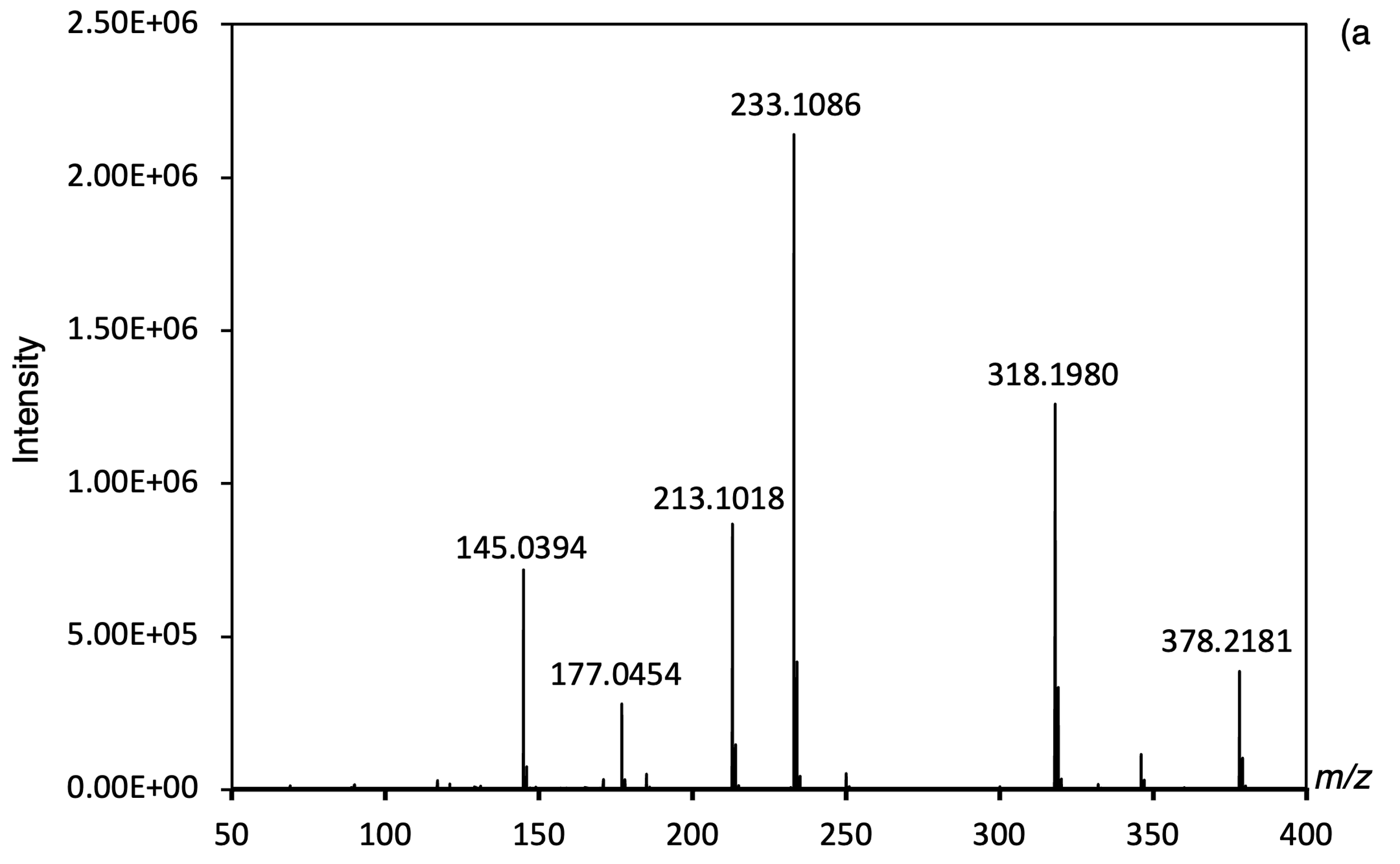


(b


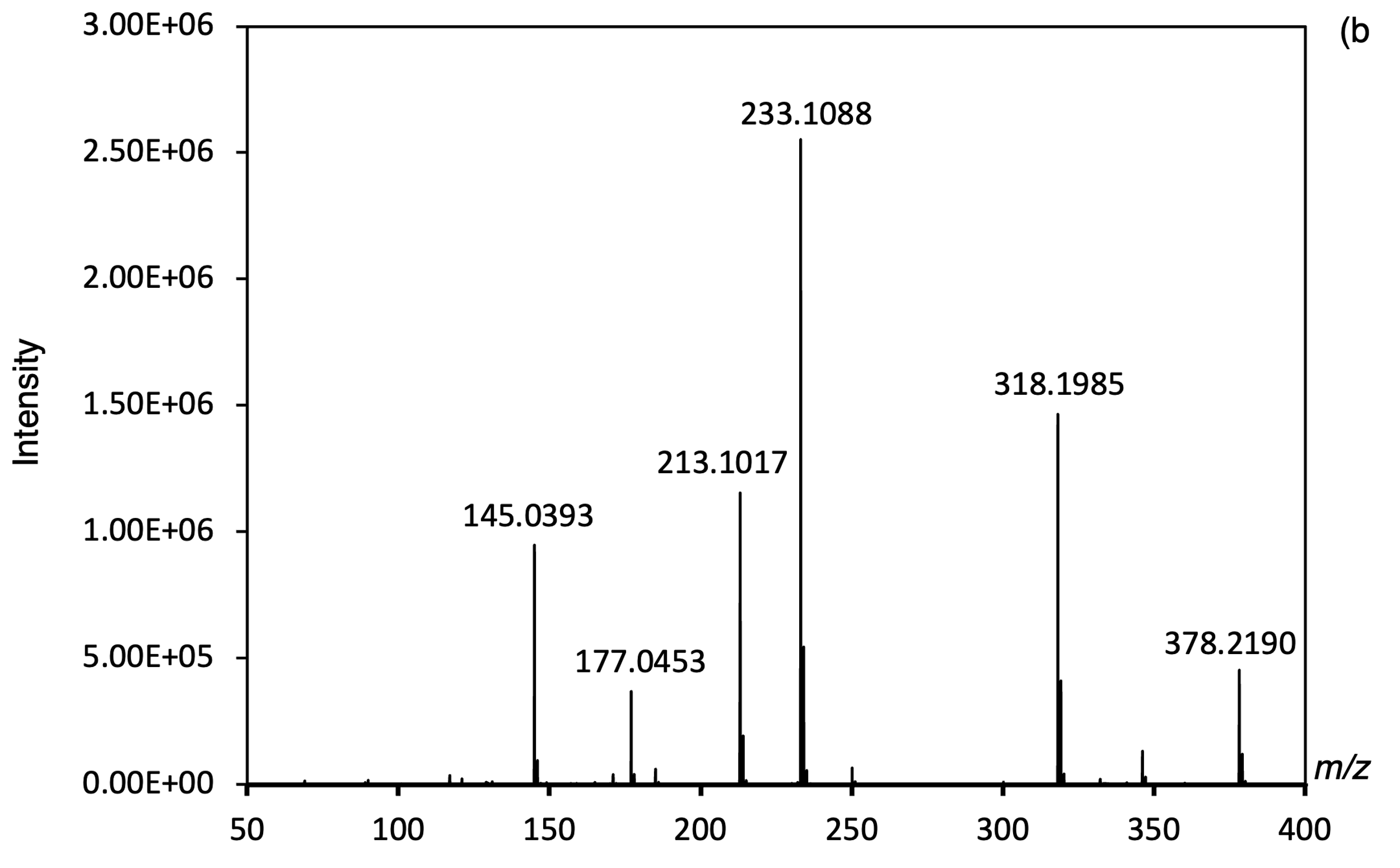


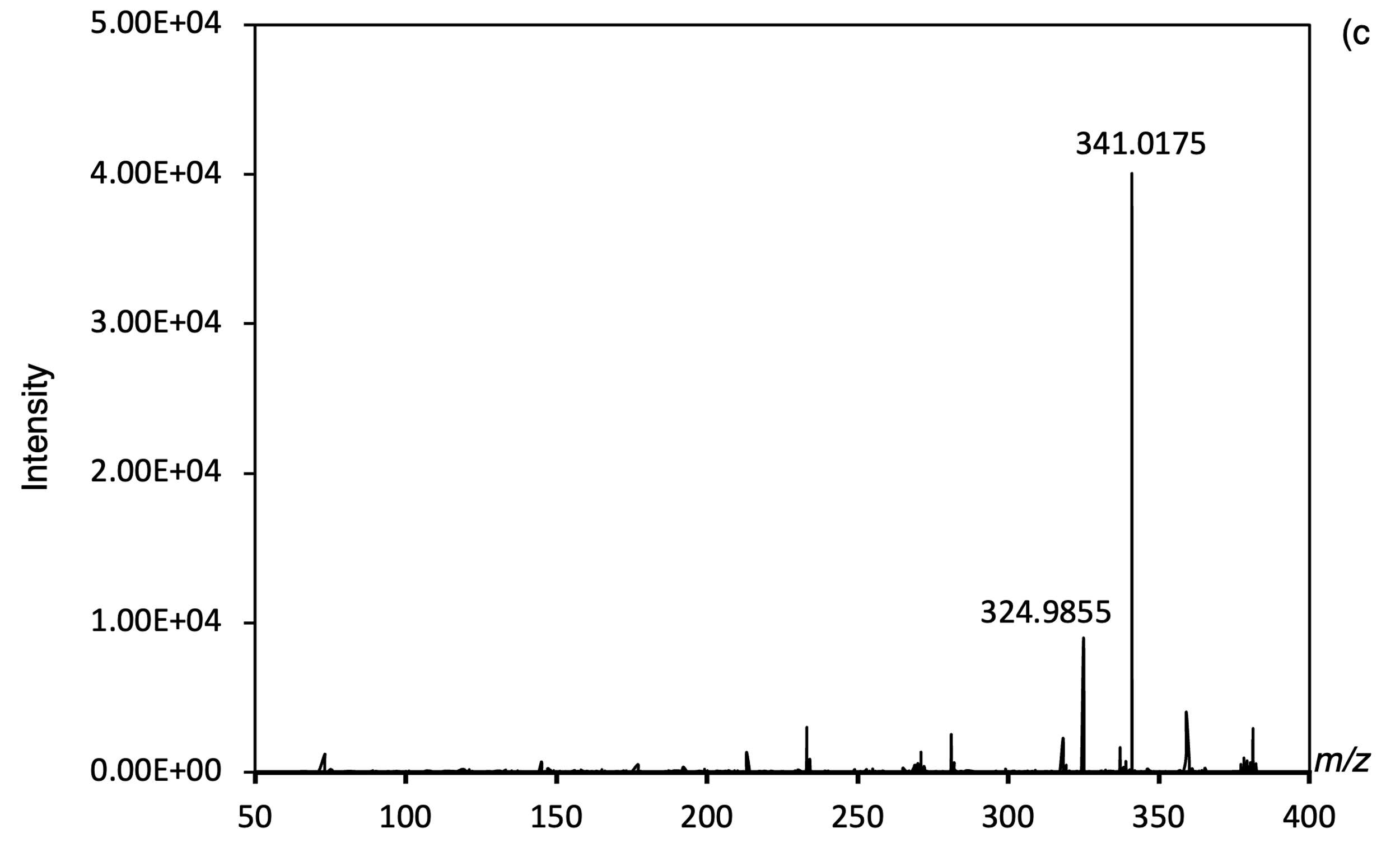


**Figure SI1.** MS of subunits 11M (a) and 5F-MDMB-PINACA RS (b) and blank paper extract in MeOH (c)

- - 1. **Method validation- calibration standards preparation**

The Stock Solution 1 (SS1) was prepared by weighting ca. 2 mg of 5F-MDMB-PINACA reference standard (RS) in a Sartorius Practum (Sartorius AS, Germany) analytical balance and adding them to a 2 mL volumetric flask. MeOH was added to reach the 2 mL final volume, to give a concentration of ca. 1 mg/mL. The SS1 was vortexed using VORTEX-GENIE2 (Scientific industries, Inc., USA) for two minutes to reach complete solubilisation. An SS2 was prepared by diluting 100 µL of SS1 into 900 µL of MeOH. The dilution scheme presented in Table SI2 was employed to prepare the 5F-MDMB-PINACA six calibration standards.

**Table SI2.** UPLC-PdA-QDa-MS 5F-MDMB-PINACA calibration standards dilution scheme.

| **Sample** | **Concentration (µg/mL)** | **Spiking volume (µL)** | | **Diluent (µL)** |
| --- | --- | --- | --- | --- |
|  |  | **SS1^*^** | **SS2^*^** |  |
| **5F-MDMB-PINACA C6** | 50 | 50 |  | 950 |
| **5F-MDMB-PINACA C5** | 20 | 20 |  | 980 |
| **5F-MDMB-PINACA C4** | 10 | 10 |  | 990 |
| **5F-MDMB-PINACA C3** | 5 |  | 50 | 950 |
| **5F-MDMB-PINACA C2** | 2 |  | 20 | 980 |
| **5F-MDMB-PINACA C1** | 1 |  | 10 | 990 |
| ^*^Stock Solution 1 concentration 1 mg/mL= 1000 µg/mL=1,000,000 ng/mL | | | | |
| ^*^Stock Solution 2 concentration 0.1 mg/mL= 100 µg/mL= 100,000 ng/mL | | | | |

The calibration standards were freshly prepared and analysed on different days.

- - 1. **Simulated paper sample analysis- preliminary evaluation of matrix effect**

The study on the paper matrix was performed to verify if other compounds present in the different types of paper could influence the final quantification of the target analyte. To this end, simulated paper samples prepared using different paper matrices were spiked with the same amount of 5F-MDMB-PINACA, which was then quantified. The matrices employed (Figure SI2) were the following: Type 1 common printing paper 80 g/m^2^ density (Envirocopy), Type 2 common printing paper 80 g/m^2^ density (Evolution), Type 3 paper from ruled notebook 80 g/m^2^ density (Pukka Pad), Type 4 weighted paper 120 g/m^2^ density (Evolution), and Type 5 black ink printed common printing paper 80 g/m^2^ density (Envirocopy).


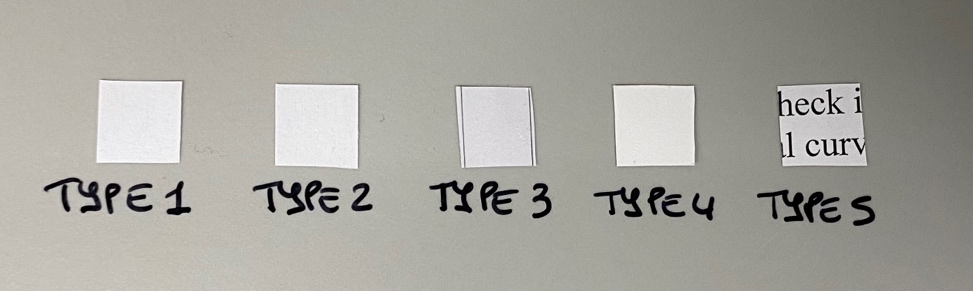


**Figure SI2** Different types of paper matrix evaluated for the study.

20 µL of a 1 mg/mL 5F-MDMB-PINACA RS solution were pipetted onto the five different paper types, extracted, and analysed using the UPLC-QDa-MS. The peak areas of each extraction were collected, quantified, and compared to each other’s using the relative standard deviation (RSD), to verify if there was any big fluctuation in the quantity of 5F-MDMB-PINACA extracted when different paper types were employed to prepare the simulated paper samples (Table SI3).

**Table SI3.** Summary of results of the five paper types evaluated in the paper matrix evaluation study.

| **Samples** | **Average AUC**  **(AU)** | **Average concentration (ng/mL)** | **Average recovery**  **(%)** | **RSD AUC**  **(%)** | **RSD concentration (%)** | **RSD recovery**  **(%)** |
| --- | --- | --- | --- | --- | --- | --- |
| **Type 1 paper** | 115057 | 17446 | 81.5 | 2.25 | 2.27 | 2.47 |
| **Type 2 paper** | 113808 | 17218 | 80.4 |  |  |  |
| **Type 3 paper** | 116676 | 17668 | 82.6 |  |  |  |
| **Type 4 paper** | 118216 | 17928 | 83.8 |  |  |  |
| **Type 5 paper** | 120807 | 17835 | 85.7 |  |  |  |

The five paper types evaluated in this study, employed to prepare the simulated paper samples spiked with 20 µL of a 1 mg/mL 5F-MDMB-PINACA RS solution did not lead to alteration of the ionisation efficiency of the target analyte quantified. The RSD between the AUC resulting from the analysis of the different types of paper matrices was 2.25%. While the RSD between the 5F-MDMB-PINACA concentration was 2.27%, and the RSD between the percentage recovery was 2.47%. To note that the data collected are limited (n=1), as no replicates were evaluated for each type of paper used, and this study only intended to estimate the spread of the results to gain insight into the behaviour of different paper matrices. The low RSD calculated over the five types of paper, suggests that no matrix effect arises when quantifying these specific types of samples. More replicates, and additional paper types should be systematically evaluated in the future to ensure statistically valid conclusions are reached.

**3.2.1 Method validation- summary results**

|  | **1^st^ calibration curve** | **2^nd^ calibration curve** | **3^rd^ calibration curve** | **4^th^ calibration curve** | **5^th^ calibration curve** | **6^th^ calibration curve** | **Acceptance criteria** |
| --- | --- | --- | --- | --- | --- | --- | --- |
| **Theoretical plate number (N)** | 2314.32 | 29743.54 | 2437.49 | 29976.98 | 2256.09 | 2896.65 | N > 2000 |
| **Tailing Factor (T)** | 1.03 | 1.01 | 1.06 | 1.01 | 1.02 | 1.05 | T < 2 |
| **Linearity (R²)** | 0.9958 | 0.9989 | 0.9988 | 0.9997 | 0.9985 | 0.9995 | R² > 0.99 |
| **Limit of Detection (ng/ml)** | 0.1076 | 0.0601 | 0.0577 | 0.0304 | 0.0639 | 0.0386 | - |
| **Limit of Quantification (ng/ml)** | 0.3261 | 0.1822 | 0.1750 | 0.0921 | 0.1937 | 0.1170 | - |
|  | **1^st^ calibration curve** | **2^nd^ calibration curve** | **3^rd^ calibration curve** | **4^th^ calibration curve** | **5^th^ calibration curve** | **6^th^ calibration curve** | **Acceptance criteria** |
| **Precision under repeatability & reproducibility condition** |  |  |  |  |  |  | % RSD < 15%  % RSD < 15%  % RSD < 15% |
| RSD % at concentration 1 (highest) | 0.85 | 0.51 | 0.16 | 0.23 | 0.59 | 0.47 |  |
| RSD % at concentration 2 | 0.69 | 0.53 | 0.55 | 0.99 | 0.5 | 0.42 |  |
| RSD % at concentration 3 | 0.42 | 0.51 | 0.33 | 0.65 | 0.46 | 0.73 |  |
| RSD % at concentration 4 | 1.09 | 1.37 | 0.99 | 0.29 | 0.36 | 0.37 | % RSD < 20% |
| RSD % at concentration 5 | 3.57 | 3.68 | 2.56 | 1.29 | 3.64 | 2.81 | % RSD < 20% |
| RSD % at concentration 6 (lowest) | 4.63 | 2.92 | 2.16 | 1.33 | 4.93 | 4.31 | % RSD < 20% |
| **Accuracy** |  |  |  |  |  |  |  |
| Accuracy % at high concentration | 109 | 90 | 109 | 91 | 88 | 102 | % Error < 15%  % Error < 15%  % Error < 20% |
| Accuracy % at medium concentration | 107 | 105 | 104 | 101 | 87 | 105 |  |
| Accuracy % at low concentration | 81 | 99 | 85 | 80 | 105 | 87 |  |

**Table SI4.** Method validation summary results
